# “A Pirate Goes Nee-Nor-Nee-Nor!” Humor With Siblings in Middle Childhood: A Window to Social Understanding?

**DOI:** 10.1037/dev0001403

**Published:** 2022-06-02

**Authors:** Amy L. Paine, Salim Hashmi, Nina Howe, Nisha Johnson, Matthew Scott, Dale F. Hay

**Affiliations:** 1 School of Psychology, Cardiff University; 2 Department of Psychology, Institute of Psychiatry, Psychology and Neuroscience, King’s College London; 3 Department of Education, Concordia University

**Keywords:** humor, pretend play, siblings, social understanding

## Abstract

Humor is a central feature of close and intimate relationships in childhood. However, fundamental questions regarding the relationship between humor production, pretend play, and social understanding have been overlooked. In a selected subsample from a prospective longitudinal study of first-born children (*N* = 110, *M* age = 6.91 years, 46.4% female, 98.1% parents identified as English, Welsh, Scottish, or Irish), we conducted detailed observational coding of children’s humor production during dress-up play with younger siblings. Focal children also completed a battery of social understanding tasks that measured emotion understanding and second-order belief understanding. Focal children were also observed during solo free play with Playmobil, and their spontaneous references to others’ cognitions and play with objects were coded. Correlation analyses indicated that children’s word play with their sibling was associated with their tendency to engage in pretense during solo play. Regression analyses showed that humorous sound play with siblings was associated with their emotion understanding and playful teasing with siblings was associated with their spontaneous references to others’ cognitive states during solo free play. Our findings contribute to knowledge and theory regarding domains of development associated with humor production in childhood.

A child’s ability to conceive and express humor is thought to harness and integrate cognitive, social, and emotional understanding (Loizou & Recchia, 2019). Humor comprises “a cognitive process and an emotional response” (Martin, 2007, p. 83); its production involves the playful violation of norms to create incongruities, for example, in manipulating meanings, language, concepts, and creating absurdities and unexpected behavior (McGhee, 1979; Mireault & Reddy, 2016; Pien & Rothbart, 1976; Schultz, 1976). Such playful incongruities often (but not always) generate laughter and, more broadly, amusement in others (Mireault & Reddy, 2016). Humor is, therefore, often social in nature; being able to perceive and express humor, and create amusement in others, is thought to require the ability to appreciate and understand the thoughts and feelings of others (Airenti, 2016; Hoicka & Akhtar, 2012; Leekam, 1991; Martucci, 2016). Children use others’ intentional cues to distinguish jokes from mistakes from two years of age (Hoicka & Gattis, 2008), and children as young as three can understand jokes and nonliteral ironic utterances (Airenti, 2016; Angeleri & Airenti, 2014; Loukusa & Leinonen, 2008). Angeleri and Airenti (2014) also reported associations between the comprehension of jokes and children’s understanding of false beliefs.

However, most studies focus on children’s perception of humor, as opposed to their creation of humorous acts (Goodchilds, 1972; Reddy, 2001). Humor production is one of the building blocks of warm and playful relationships (Dunn, 1988; Paine, 2021) that develops in close connection with children’s play and emerging social understanding (Bergen, 2002, 2019; Dunn, 1988), but very little research has investigated associations between children’s use of humor in their close relationships and other domains of child development (Bergen, 2019). Therefore, we investigated 7-year-olds’ spontaneously produced humor during play with their siblings in relation to pretend play and social understanding skills.

## Humor Production in Childhood

According to McGhee (1979, 2002), the development of children’s humor can be defined in stages in their emerging ability to recognize and produce incongruities. By the end of the first year of life, most infants intentionally produce humorous acts that are responsive to the behavior and emotional reactions of others, for example, clowning (e.g., producing odd movements, facial expressions, actions, and vocalizations) and teasing (e.g., pointing to an object and then refusing it) to elicit laughter from their caregivers (Reddy, 2001; Reddy & Mireault, 2015). Such humorous actions are often discovered by accident; following an unintentional incongruity that made someone laugh, infants repeat the act deliberately to elicit the same reaction (Mireault & Reddy, 2016).

From the second year, children begin to produce novel humorous acts, including incongruous language, such as chanting and creating nonsense words (e.g., “goo-joo-boo-joo”; Hoicka & Akhtar, 2012; Loizou, 2005). By middle childhood (around age 7), children produce more complex conceptual incongruities and word play, such as comedic hyperbole, puns, and riddles (e.g., “Whaddya call a test tube with a college degree? A graduated cylinder!”; Bergen, 2002, 2012; Paine, Hashmi, et al., 2019; Varga, 2000). Evidence demonstrates that children’s repertoire of humor expands across development (Bergen, 2002); incongruous actions (e.g., piling toy animals on a toy farm roof), physical play (e.g., silly posing, dancing, and facial expressions), sound play (e.g., shouting in a high-pitched voice, “Ew! I’ve been slimed!”), playful teasing and banter (e.g., “Ya dummy!”), and breaking taboos (e.g., “H, I, J, K, L, M, N, O… PEE? Get it? Pee!”) are all common forms of humor produced by 5-to-7-year-olds (Paine, Howe, et al., 2019; Paine et al., 2021).

## Connections Between Humor, Play, and Social Understanding

The ability to create humor is thought to be closely linked with children’s developing ability to understand the minds of others (Bariaud, 1989; Bergen, 2002; Dunn, 1988; Leekam, 1991; Martucci, 2016). Children demonstrate an understanding of the social world through shared humor in their early interactions, long before they have developed a fully-fledged theory of mind (Addyman & Addyman, 2013; Airenti, 2016; Mireault et al., 2011, 2014; Reddy & Mireault, 2015; Sroufe & Wunsch, 1972). There is considerable evidence that awareness of others’ intentions, expectations, and beliefs emerges as early as 7 months of age (Hamlin et al., 2007; Kovács et al., 2010). Clowning, teasing, and other “communicative games” in infancy and early childhood may require understanding of the minds of others (as argued by Mireault & Reddy, 2016), even if the incongruous acts themselves are not understood in a deep way (Airenti, 2016; Bergen, 1998; Bergen, 2019). In line with social interactionist theories of development, humor during play may, therefore, act as a “melting pot,” providing a context for rehearsing existing social understanding skills and in turn, drive development and new competencies (Gibson et al., 2020; Lillard et al., 2013).

Theorists have long hypothesized a close partnership between humor and play (Bariaud, 1989; Bergen, 2012; Bergen, 2019; Wolfenstein, 1954). Both are enjoyable, internally motivated, and involve distortions of reality (Bergen, 2012). Some researchers have identified the importance of humor in the personality construct of playfulness. Indeed, according to Barnett (2007) playfulness is defined as “… the predisposition to frame (or reframe) a situation in such a way as to provide oneself (and possibly others) with amusement, humor, and/or entertainment” (p. 955). Recent evidence demonstrates that 5-to-7-year-olds’ self-perceptions of their tendencies to tell jokes and funny stories, do silly things, and make others laugh contributes to their perceptions of their own playfulness (Fink et al., 2020). In particular, childhood humor and pretend play may be underpinned by similar developmental processes, where both playful activities are argued to involve symbolic thinking (McGhee, 1979; Rubin et al., 1983; Tower & Singer, 1980). Sharing humor requires the “performer” to understand and convey to their “audience” that their playful, nonliteral acts are of a “pretend,” and not an “incorrect” nature (Bateson, 1955; Bergen, 2012; Leekam, 1991). But unlike some pretend play, children’s humor involves the creation of alternative or distorted realities through silliness or nonsense, with the goal of triggering amusement (i.e., the distinction between “joking pretend” and “serious pretend”; Bariaud, 1989; Wolfenstein, 1954).

Although evidence is mixed (for a review, see Lillard, 2013), many researchers have proposed that pretend play involves an understanding of one’s own and others’ mental states (Weisberg, 2015). Leslie (1994, 1987) proposed that in pretend play, a child mentally projects a nonliteral reality onto a situation, all the while knowing what the real situation is. For example, when a mental representation of a telephone is projected on a banana, a child simultaneously appreciates two contradictory models of reality (Lillard, 1993). The same mental capacity, it is thought, is necessary for understanding when someone holds a belief that differs from one’s own (i.e., holds a false belief, Leslie, 1994, 1987). For example, Harris (2000) proposed that pretend play involves social understanding skills, insofar that in pretend play, children simulate mental states they do not actually experience and states of the world that are not reality.

Sociological communications perspectives suggest that both shared pretend play and humor depend on children’s ability to understand that their partners share their intent to be playful (Bariaud, 1989; Bergen, 2019; Semrud-Clikeman & Glass, 2010), as well as their ability to communicate ludic cues that “this is play” (i.e., smiling, laughter, sound effects, and exaggerated actions), which usually occur in the play frame (Bateson, 1955; Bergen, 2019). In common with pretend play, producing humor successfully may depend on a child’s social understanding. Without a frame of mutual playful intent and complicity, incongruities might confuse or even threaten children’s play partners (Semrud-Clikeman & Glass, 2010).

The theoretical and conceptual links between humor, pretend play, and social understanding are clear. However, most contemporary developmental research on humor has focused on describing children’s humor in play, rather than investigating associations between humor and other domains of child development (Bergen, 2019). Is a child’s tendency to spontaneously produce humor associated with their tendency to engage in pretense? We examined this theoretical partnership by investigating children’s spontaneous humor production during social play with their use of objects in solo play with toys, distinguishing between (a) handling and setting up props; (b) transforming objects in expected ways (e.g., animating a horse to gallop); and (c) transforming objects in creative ways (e.g., animating a cow to fly; Howe et al., 2014). Insofar as transforming objects in pretend play requires symbolic, creative thinking (Piaget, 1962)—thought also to be required for humor production (Bariaud, 1989; Wolfenstein, 1954)—we were interested in associations between children’s tendency to produce humor and their creative object transformations during play.

In our previous work, we found that 5-year-old children’s spontaneous humor production was positively associated with their tendencies to talk about thoughts and knowledge (i.e., cognitive states) during play (Paine et al., 2021). We now aim to expand knowledge about the association between children’s humor production during social play and their developing understanding of minds in middle childhood, using a battery of age-appropriate tasks that measure 7-year-olds’ social understanding. If producing humor successfully in middle childhood depends on children’s knowledge that their partners will be able to represent their own inner states and decouple reality from make-believe, we would expect that having better insight into the beliefs embedded within their partner’s beliefs (i.e., second-order social understanding) would be positively associated with humor production. Therefore, we hypothesized that humor production would be associated with children’s understanding of second-order false belief (Coull et al., 2006; Perner & Wimmer, 1985; Paine, Hashmi, et al., 2019).

In middle childhood, children increasingly refer to other people’s cognitive states (i.e., thoughts and knowledge), which is considered an index of children’s advanced understanding of the minds of others (de Rosnay & Hughes, 2006) and is associated with activation in brain areas associated with social processing (Hashmi et al., 2022). Sharing humor successfully may require children to appreciate the mental states of others to understand that their play partners will find their humorous acts to be amusing (Bergen, 2019; Leekam, 1991). Interpreting mental states, such as nonverbal emotional displays, presents different cognitive demands than talking about the mental states of others (Harris et al., 2005; Ruffman, 2014). However, a key feature of successful humorous exchanges is that both interactional partners have mutual complicity in the play frame (Semrud-Clikeman & Glass, 2010); thus, a child’s ability to interpret and understand emotional cues may be an important correlate of humor production. We therefore hypothesized that children’s humor production would be positively associated with their tendency to talk about the cognitive states of others and their ability to perceive and understand others’ emotions.

## Sibling Interaction as a Context for Humor Production

Siblings share a long, co-constructed history (Dunn, 2007; McHale et al., 2012) that makes their relationship a particularly important context for the development of social understanding (Howe & Recchia, 2014; Howe et al., 2022) and humor (Dunn, 1988; Paine, Howe, et al., 2019; Paine et al., 2021). In our previous work, we have observed that children’s humor produced with siblings often appears to be well-rehearsed, ritualistic, and based on the siblings’ intimate knowledge of, and experiences with, one another (Paine, Howe, et al., 2019; Paine et al., 2021). Siblings who share day-to-day family life are highly familiar with one another, which can result in emotionally intense and uninhibited interactions (Howe & Recchia, 2014). With siblings, children often engage in playful, mischievous behavior (Paine, Howe, et al., 2019; Paine et al., 2021), such as taboo-themed humor and playful teasing (e.g., “Where’s your bum brain?”), as well as word and sound manipulations (e.g., chanting, “Chocolatina, chocolatina!”) and incongruous actions (e.g., playfully putting their foot on the sibling’s head). Given that sibling interactions are a rich context for humor production, we have now investigated children’s spontaneous production of humor during dress-up play with a younger sibling.

## The Present Study: Aims and Hypotheses

A child’s developing ability to perceive, interpret, and understand the minds of others is a vital skill in being able to navigate the social world (Carpendale & Lewis, 2015). Although the ability to conceive and express humor is likely to be associated with a sophisticated understanding of minds (Bergen, 2002; Dunn, 1988; Leekam, 1991), few studies have tested this association (Bergen, 2019; Paine et al., 2021). We sought to contribute to developmental theory that suggests childhood humor and pretend play may be underpinned by similar developmental processes (Bergen, 2019) and that a child’s production of humor may reflect their social–cognitive development (Bariaud, 1989; Bergen, 2002; Dunn, 1988; Leekam, 1991). Therefore, we drew on a corpus of observational data of interactions between siblings during play from a larger study of the development of first-born children (Hay et al., 2021). As part of a home visit at age 7 years, we conducted detailed observational coding of the focal children’s humor production during dressing-up play with younger siblings. Focal children also completed a battery of social understanding tasks to assess emotion and second order belief understanding. This battery of tasks included a solo free play task with Playmobil toys, which was coded for children’s engagement in pretend play and tendency to talk about the cognitive states of others.

Our first overarching aim was to describe the humor that was spontaneously produced during play with a sibling. Previous evidence suggests that humor shared with siblings is influenced by sibling constellation factors (i.e., birth order and gender composition). In early childhood, first-born children tend to produce more types of humor with their siblings—including playful banter and teasing and action and object incongruities—than do second-born children of the same age (Paine et al., 2021). In middle childhood, pairs of brothers produce more humor overall than pairs of sisters (Paine, Howe, et al., 2019). Similarly, children’s social understanding skills are influenced by sibling constellation factors (e.g., age gap; Paine et al., 2018). The current analyses focus on the community sample of first-born 7-year-olds, which precluded analysis for the effects of birth order that would not be confounded with age. However, we did examine the 7-year-olds’ humor production in relation to the age gap between siblings and the gender of the second-born child. Given that closer-in-age children tend to have fewer developmental asymmetries and more mutual interests (Hughes, 2011), we hypothesized that siblings with a smaller age gap would produce more humor. In line with previous work (Paine, Howe, et al., 2019), we hypothesized that play between two brothers would produce more humor than other gender combinations.

The second overarching aim was to investigate associations between the focal children’s humor production during sibling play and other skills, in particular their engagement in pretend play and performance on social–cognitive tasks. Given the close theoretical partnership between humor and pretend play (Bergen, 2019), we expected that children who play with objects in creative ways would also produce more humor. We also expected that humor production would be positively associated with social understanding skills, as indicated by children’s emotion understanding, tendency to talk about others’ cognitive states, and their understanding of second-order beliefs (Bergen, 2019; Dunn, 1988; Leekam, 1991; Martucci, 2016; Paine et al., 2021). Given that any significant associations with social understanding could be explained by known covariates (i.e., child age, language ability, working memory, family demographics; Cole & Mitchell, 2000; Lecce et al., 2017; Paine et al., 2018), we controlled for these covariates.

## Method

### Participants

The present study focuses on *N* = 110 first-born children, a selected subsample of those who had been recruited for a longitudinal study of child development during their mothers’ pregnancies (the Cardiff Child Development Study; Hay et al., 2021). The focal children were observed at home when they were between 6.5 and 7.5 years of age (*M* age = 6.91 years, *SD* = .38); 51 (46.4%) were female. Sociodemographic characteristics of the sample are presented in Table 1. The longitudinal sample of first-borns had been recruited from National Health Service (NHS) prenatal clinics in hospitals and GP surgeries in Cardiff and Vale University Health Board, and the Gwent Health care Trust, U.K. Ethical approval for the Cardiff Child Development study was obtained for the procedures from the NHS Multi-Centre Research Ethics Committee and the Cardiff University School of Psychology Research Ethics Committee.[Table tbl1]

Progression to the current sample of 110 participants was as follows: 332 first-time mothers were recruited into the study during the 3rd trimester of pregnancy. Of the 332 families who joined the study, 321 (97%) participated in the study after the child’s birth, and 287 (89%) families took part in the 6.5- to 7.5-year visit, with 271 observed at home and the others completing questionnaires. At this time point, 172 children had at least one younger sibling living with them in the home; however, not all siblings could be present at the time of the observation. Forty-nine focal children were not observed in the sibling play session, due to the sibling being too young to join in the play, being absent from home at the time of the assessment or refusing to take part in the observation. Two cases had technical errors in recordings; six videos were not codable (very short, chaotic, or with no translation of the children’s speech available). In five cases, the sibling play session was not completed.

The focal children were observed with their closest-in-age younger sibling, but other younger siblings were allowed to join the play if they wished. Therefore, 95 7-year-olds (86.4%) were observed in play with one younger sibling, 14 (12.7%) with two younger siblings, and one (.09%) with three younger siblings. The *M* age of the next-in-age sibling was 4.55 years (*SD* = 1.01), range 2.08 to 7.42 years; 54 (49.1%) were female. The sibling play sessions included 34 (30.9%) brother pairs, 29 (26.4%) sister pairs, 22 (20.0%) older sister with younger brother pairs, and 25 (22.7%) older brother with younger sister pairs. Six siblings (5.5%) were twins of the focal child, 100 (90.9%) were full siblings and 10 (9.1%) were half siblings.

### Procedure

Families were visited in the home by research assistants for two two-hour sessions (*M* age 6.91 years at session 1; 6.98 years at session 2). A questionnaire battery was provided during the visit to primary caregivers and, where possible, fathers. While the primary caregiver completed an interview with one trained research assistant, the child completed various cognitive, social, and emotional assessments in a quiet space with a second trained research assistant. At the end of the second session, children completed the dress-up task with younger siblings who were at home and consented to join the play. The child and caregiver then participated in a parent–child interaction task and some family games. When required, a third research assistant accompanied the research team to keep younger siblings from interfering with the child testing and parent–child interaction tasks. A remuneration of £20 was given to the caregiver and a book voucher of £10 to the child at the end of the home visits.

### Measures

#### Family Background

At the time of recruitment into the study, information about fathers was not available for all cases; therefore, the sociodemographic variables used to characterize each family in the sample were based on data collected from mothers at entry to the study in pregnancy. These variables were: (a) social class, assessed using the U.K. Standard Occupational Classification 2000 (SOC2000; Elias et al., 1999) to determine maternal occupational status. This measure was based on the highest ranked employment that the mother ever had at entry into the study. A dichotomous variable was created using mothers’ highest rank of employment on the SOC2000 six-category scale to categorize individuals as working class or middle class. (b) Maternal education, a dichotomized variable indicating whether mothers had achieved the minimum level of qualifications required for the completion of secondary education in the United Kingdom (General Certificate of Secondary Education examinations grade A*-C or equivalent).

#### Children’s Receptive Vocabulary

Children’s vocabulary knowledge was assessed using the British Picture Vocabulary Scale (BPVS; Dunn et al., 1982). In this task, the experimenter spoke a word to the child, who was asked to point or say the number of the picture that corresponded to the word. Each child’s receptive vocabulary score was computed by age normalizing the data to produce a standardized score. All children in the subsample completed this assessment.

#### Children’s Working Memory

Children’s working memory was assessed using a task from the Amsterdam Neuropsychological Tasks (ANT), (de Sonneville, 1999). The visuo-spatial sequencing (VSS) task measured children’s visuospatial working memory. Children were presented with a gray square containing nine circles symmetrically positioned in a 3 ×3 matrix on a computer screen. After a beep, a sequence of circles was pointed at by a computer animated hand, and after the sequence children took control of the mouse to replicate the sequence of circles. The test consisted of 24 trials and gradually increased in difficulty in the number of targets and complexity of the sequence. Working memory was assessed using the total number of correct targets in the correct order, with a total of 100 possible correct targets. Data were available for 105/110 (95.5%) families in the subsample.

#### Children’s Second-Order False Belief Understanding

Children were told a second-order false belief story to assess their higher-order understanding of belief (Paine et al., 2018; Paine, Hashmi, et al., 2019). This age-appropriate task was adapted from the second-order belief paradigms used by Coull et al. (2006) and Perner and Wimmer (1985). Children were told a story by the tester, enacted with Playmobil figures. In the story, the protagonist (Nick) shows his special teddy to the child and tucks the teddy inside the bed. The mother comes into the room and asks Nick to brush his teeth; they leave the room. In Nick’s absence, the sibling (Alex) removes the teddy from the duvet and hides the teddy in the cupboard. Unbeknownst to Alex, Nick returns and watches Alex hiding the teddy, before leaving the room again. When Nick comes back into the room he says, “I want my teddy.” Children had to answer the belief (“Where does Alex think Nick will look for the teddy?”), justification (“Why does Alex think Nick will look for the teddy in the [child’s answer]?”), and comprehension questions (a. “Does Nick know that the teddy is in the cupboard?”; b. “Does Alex know that Nick saw her hide the teddy?”; c. “Where will Nick look for the teddy?”) correctly to be classified as passing second-order false belief. Data were available for 108/110 (98.2%) children. Excellent reliability was established for passing this task (κ = 1.00, see Paine et al., 2018).

#### Child’s Emotion Understanding

Children’s emotion understanding was assessed using valid and widely used measures that assess emotion recognition and emotional perspective-taking (Denham, 1986; Lane et al., 2010). First, children were asked to match eight faces printed on a card that depicted different emotions to four target emotions (van der Schalk et al., 2011). The experimenter asked, “Can you find someone who is [scared x2/cross x2/happy x2/sad x2]?” Each correct match of a label to the emotion face was scored as one, yielding a total possible score of eight.

Second, an adapted version of an emotional perspective-taking task was administered (Pollak et al., 2000). Children were presented with a new card showing four emotions from the card in the previous task that were gender-matched to the participant. Children were told short vignettes in one of two counterbalanced orders, in which the protagonist would experience happiness, sadness, anger, or fear. The protagonist in the stories was gender-matched to the child. Following each vignette, the child was asked to repeat the story to a puppet to demonstrate their understanding, and then to indicate how the protagonist would feel by pointing at one of the four presented emotion faces. Children earned a score of one for every correctly identified emotion per vignette, yielding a possible score of eight. The accuracy scores for the emotion recognition and emotional perspective-taking tasks were combined and transformed into percentage of correct scores as a measure of children’s emotion understanding (Lane et al., 2010). Data were available for 109/110 (99.1%) children.

### Observational Coding

#### Children’s Use of Objects During Solo Play

Following the battery of social–cognitive tasks, children were video recorded as they freely played with Playmobil figures for 3 min with the experimenter present, who was instructed not to prompt or participate in the children’s play. A transcript of children’s physical behavior and use of objects during the play was made alongside their speech in 5-s time segments. Use of objects during play was coded using a scheme developed by Howe et al. (2014), and included (a) no use of object; (b) handling of object (i.e., holding, handling, examining objects); (c) set up/organization of object (i.e., physically setting up but not using objects in play); (d) expected use of object (i.e., using/animating the object in a conventional way given the form and function of the object); (e) creative use of object (i.e., changing the identity or function of an object in a way that is not typical of that object in reality). Excellent interrater reliability was established (median ICC = .97). Data were available for 105/110 (95.5%) children in the present subsample.

#### Children’s Speech During Solo Play

Children’s speech during the Playmobil free play task was coded for children’s references to internal states, which included seven categories: perception, physiology, preference, intention, desire, emotion, and cognition. All categories were additionally coded for the referent of the speech about inner states (i.e., about the child’s own states, states of others [i.e., the Playmobil characters]). Excellent interrater reliability was established (see Hashmi, Paine, et al., 2021; Paine, Hashmi, et al., 2019; median ICC = .95).

A measure of children’s talkativeness was also computed for this task by dividing the number of 5-s segments by the total number of segments in the task to yield a proportional score between 0 and 1. Data were available for 106/110 (96.4%) children in the present subsample (one family had an audio recording only).

#### Children’s Humor With Siblings in a Dressing-Up Task

In Visit 2 of the home observations, focal children were observed as they played with their younger sibling(s). Children were presented with a box containing plastic tea set items (teapot, cups, plates, and play food) and a set of hats (witch, chef, builder, pirate, police, crown, and sunhat). The experimenter presented the children with the toys and hats, and said, “Now we’re going to play dress-up. I’ve got some hats. We can be different people with these hats. Which hat do you want? Who would you like to be?” Children were then allowed to pick a hat and play freely for a 5 min video-recorded session. Experimenters were instructed not to prompt the children’s play and only participated in the play at the children’s request.

First, both the focal child and their sibling’s laughter was coded using 10-s interval time sampling across the 5-min dressing up task. Two independent observers identified intervals of the videos that contained laughter in 20 (18.2%) of the cases with good agreement (κ = .91). Children’s language and behavior within each interval containing laughter was transcribed by one observer and double-checked by a second observer.

After the children’s language and behavior were transcribed, we used both the transcripts and video records to code where children’s humor was the source of laughter. This included humor that resulted in laughter by the play partner and humor where the child producing the humorous act laughed themselves. This scheme included seven categories of humor (Paine, Howe, et al., 2019; Paine et al., 2021; see Table 2). A random sample of 23 (20.9%) play sessions was coded independently for categories of humor; interrater reliability was excellent (κ = .93), and disagreements were resolved via consensus between coders.[Table tbl2]

### Data Analysis

We first describe focal children’s humor production during the dressing-up play session with their sibling. To control for slight variability in length of the play session videos (due to bathroom breaks, interruptions, etc.), all coded variables were prorated, by dividing each variable by the length of the interaction and multiplying by the target interaction time. We then report our examination of differences in focal children’s humor production as a function of characteristics of the sibling relationship and mutual influences between siblings. Given the skewed nature of this type of observational data, we log-transformed all humor category variables to more closely approximate a normal distribution and to limit the range of scores.

Next, to identify potential covariates, we examined associations between focal children’s total humor production and potential family and within-child variables (i.e., family background, child age, receptive vocabulary, working memory). We examined associations between focal children’s humor production (total and within categories) and their pretend play with objects during solo play. As creative use of objects occurred rarely, this category was combined with expected use of objects and log-transformed to address skewness.

Finally, we examined associations between focal children’s humor production and markers of their social understanding (second-order false belief, emotion understanding, and references to others’ cognitions during solo play). Children’s references to others’ cognitions during solo Playmobil play were dichotomized for analysis, due to a high frequency of zero scores and a skewed distribution.

An iterative approach was taken to examine associations: Associations that reached *p* < .05 were followed up with regression analyses where appropriate, controlling for identified family and within-child correlates of children’s humor production and social understanding tasks. Analyses of variables entered into regression models showed no collinearity (variance inflation factor < 10, tolerance > .20; Myers, 1990). A post hoc power analysis for linear regression with three predictors was conducted using G*Power (Faul et al., 2009), which indicated that a sample of 110 participants yielded statistical power at .93 to detect small to medium effect sizes *r* = .15 at an alpha level of .05. This study was not preregistered. Data are available from the corresponding author upon request.

## Results

### 7-Year-Olds’ Humor With Younger Siblings

Descriptive statistics for humor produced by the focal child are presented in Table 3 and associations between humor categories are presented in the online supplementary materials. Most focal children produced at least one instance of humor (75.5%). The most common type of humor was performing incongruities, where, for example, children stacked different hats on their head in a tower, piled the play food on the hat they were wearing, or drank directly out of the spout of the teapot. Other common categories included preposterous statements or humorous anecdotes, for example, announcing, “A pirate goes nee-nor-nee-nor!,” and playful teasing; for example, one child said in a playful voice, “[Sibling’s name] is a worm, [Sibling’s name] is worm juice!” to which both children laughed. Categories of humor would often co-occur within the same humorous act. For example, one focal child performed an incongruity while producing sound play: wearing the witch hat, the child playfully bounced the plastic knife and fork from the tea set on the younger sibling’s head, while singing, “choppy-chop-chop!” in a sing-song voice, followed by laughter from their sibling (see Table 2).[Table tbl3]

We investigated whether focal children’s humor differed as a function of the structural features of the sibling relationship. A series of independent samples *t*-tests showed no differences in humor production (for the total score or individual categories) as a function of the focal child’s gender (all *p*s > .08). We found no association between the number of siblings present and the focal children’s humor production (total or categories) in the play session (all *p*s > .08). We next examined focal children’s humor production according to sibling constellation factors with their closest-in-age sibling. A series of one-way ANOVAs were used to examine whether focal children’s humor production (total or categories) differed as a function of sibling gender composition (brother-brother, sister-sister, older brother-younger sister, or older sister-younger brother); these analyses showed no differences in humor production between groups (all *p*s > .19). There were no significant associations between focal children’s humor production and the sibling age gap (all *p*s > .10).

Finally, we investigated the dyadic nature of focal children’s humor by examining the associations between focal children’s total humor production and total humor produced by their next-in-age younger sibling. Sixty-four (58.2%) younger siblings produced humor, and although they produced humor less often than the focal child *t*(109) = 3.65, *p* < .001 (*M* = 2.64, *SD* = 3.79), younger siblings’ humor production was highly associated with humor produced by focal children, ICC = .79, *p* < .001. This confirms our observations that humor was often reciprocal and co-constructed in humorous sequences. For example, in one humorous exchange, the younger sibling pulled the focal child’s pirate hat off their head and threw it across the room. Laughing, the focal child then pretended to pour water into a teacup and motioned to throw its contents into the sibling’s face. The sibling then ran to put on the witch hat, picked up the teapot, and shouted, “Pour! Pour-pour-pour-pour!” while pretending to pour the contents of the teapot on the focal child. The focal child then fell to the ground dramatically as they both continued to laugh.

### Other Family Factors and Within-Child Correlates of Humor Production

To examine variables associated with humor production, our subsequent analyses focused on the focal child. We investigated within-child and family factors associated with children’s overall tendency to produce humor during play with their sibling. We first investigated children’s total humor in relation to family background variables (mother’s education and social class). We did not detect any significant associations between these family background variables and focal children’s total humor production, *r*(110) = .05, *p* = .57 and *r*(110) = .00, *p* = .99, respectively (see online supplementary materials).

We next investigated associations between within-child factors and the focal children’s humor production. We found no associations between focal children’s age and total humor production, *r*(110) = .02, *p* = .82. In terms of receptive vocabulary, the mean score for children’s receptive vocabulary was 99.06 (*SD* = 11.35), and the average age equivalent for children in the sample was 6.89 years (*SD* = 1.14) and ranged from 4.75 to 9.42 years. Receptive vocabulary was also not significantly associated with children’s humor production, *r*(110) = –.11, *p* = .26. Working memory (*M* score for correct targets in the correct order was 65.64, *SD* = 17.53) was also not significantly associated with children’s humor production, *r*(105) = –.05, *p* = .65 (see online supplementary materials).

### Is Humor Production During Sibling Play Associated With an Independent Measure of Pretend Play?

Given the close conceptual overlap between children’s humor and pretend play, we next investigated associations between children’s humor with their sibling and their tendency to engage in pretense during solo play. All 105 children who were observed during the free play task with Playmobil set up objects at least once during the task (*M* = 21.67, *SD* = 6.90). Most children pretended with the toys in expected ways (65/105, 61.9%, *M* = 4.07, *SD* = 5.92), with only five children playing with the toys in creative ways (5.8%, *M* = .12, *SD* = .76); as such, pretense in expected and creative ways were summed for further analysis. Associations between children’s tendency to set up objects and pretend in expected and creative ways and humor production are shown in Table 4. Children’s word play with their siblings was associated with the tendency to use objects in pretense in both expected and creative ways during solo play *r*(105) = .23, *p* = .02.[Table tbl4]

### Is Humor Production Associated With Social Understanding?

Finally, we tested the hypothesis that children’s humor production would be positively associated with children’s performance on a battery of social understanding tasks.

#### Second Order False Belief

Of the 108 children who completed the second order false belief task, 42 (38.9%) passed. Associations between children’s performance on the second order false belief task and their humor production is shown in Table 4; no significant associations were detected between these variables, all *p*s > .14.

#### Emotion Understanding

The mean performance on the emotion understanding task was 89.68 (*SD* = 12.63, range = 43.75 to 100). Associations between children’s performance on emotion understanding and humor production are shown in Table 4. Children’s production of sound play with their sibling was associated with their emotion understanding, *r*(109) = .22, *p* = .02. This association was further examined while controlling for identified within-child correlates of emotion understanding, including receptive vocabulary, *r*(109) = .29, *p* = .002, and working memory, *r*(104) = .27, *p* = .006; see online supplementary materials). Receptive vocabulary and working memory were not associated with children’s sound play, but emotion understanding was significantly associated with focal children producing more sound play during sibling play, β = .24, *p* = .02; however, the model overall was not significant, *F*(3, 100) = 1.86, *p* = .14, adjusted *R*^2^ = .02 (see Model 1, Table 5).[Table tbl5]

Children’s references to others’ cognitions in solo play with Playmobil. Of children who were recorded during solitary free play with Playmobil figures, 24 of 106 (23%) produced at least one reference to others’ cognitive states during solo play. Children’s speech about others’ cognitive states while playing alone with Playmobil was associated with their production of sound play, *r*(106) = .19 *p* = .05, and playful teasing, *r*(106) = .26, *p* = .008, during sibling play (see Table 4).

To check that these associations were not driven by children’s tendency to be talkative during the solo Playmobil play task, we controlled for the focal child’s talkativeness during solo play. In an initial regression, neither talkativeness nor references to others’ cognitions were associated with sound play, *p*s > .08 (see Model 2, Table 5). In a second regression, talkativeness was not associated with children’s playful teasing, but those children who referenced the cognitive states of others during solo play were more likely to engage in playful teasing with siblings, β = .22, *p* = .03, *F*(2, 103) = 4.13, *p* = .02, adjusted *R*^2^ = .06 (see Model 3, Table 5).

## Discussion

In this study, we conducted detailed observational coding of children’s spontaneously produced humor during episodes of play with a younger sibling where laughter occurred. We set out to describe children’s humor production and how it differed according to structural characteristics of the sibling relationship (i.e., age gap, dyad gender composition). We also investigated theoretically predicted associations between children’s humor production and their tendency to engage in pretense and their social understanding skills. As in previous studies (Paine, Howe, et al., 2019; Paine et al., 2021), most children produced humor with their sibling during play. Children most often performed incongruities, shared preposterous statements or humorous anecdotes, and engaged in playful teasing. This is in line with theories stating that children begin to understand and produce more linguistically complex forms of humor in middle childhood (McGhee, 1979, 2002), yet our detailed observational coding of naturalistic play also shows that 6- to 7-year-olds still enjoy producing some of the simplest and earliest forms of humor, such as incongruous actions with objects (Bergen, 2002; Paine, Howe, et al., 2019; Paine et al., 2021).

### Children’s Humor, Pretend Play, and Social Understanding

This study provides further evidence for theoretical perspectives that suggest childhood humor and pretend play may be underpinned by similar developmental processes (McGhee, 1979; Rubin et al., 1983; Tower & Singer, 1980) and share a connection with indices of social understanding. We detected associations between categories of children’s humor with their sibling and independent measures of their social–cognitive skills: their engagement in pretense, emotion understanding, and spontaneous references to others’ cognitive states during solo play with Playmobil toys. We found that children’s production of humor with their siblings—specifically, their word play—was positively associated with their tendency to engage in pretense when playing alone, supporting the view that humor and pretend play are related constructs (Bariaud, 1989; Bergen, 2019; Wolfenstein, 1954) and potentially both provide insights into a child’s tendency to be playful (Barnett, 2007; Fink et al., 2020). Given that the children’s humor was associated with their tendency to animate and transform toys during solo pretend play, but not with their tendency simply to set up the toys, this finding aligns with previous claims that both humor and pretend play are characterized by the creation of alternative or distorted realities (Bariaud, 1989), or, engagement in an “as if” stance (Garvey, 1977; Leslie, 1987; Rubin et al., 1983; Tower & Singer, 1980).

However, not all types of humor, nor humor production overall, were associated with the children’s tendency to engage in pretense in solo play, suggesting that, although some characteristics of humor and pretend play appear connected, they are still distinct forms of playfulness. Researchers have emphasized the difference between humor and pretense as different forms of make-believe; for example, when joking, children emphasize nonsense incongruities, but when engaging in “serious” pretense, they create imaginary worlds that make sense (Bariaud, 1989; Wolfenstein, 1954). Although our findings provide some insight into the nature of the connectedness of humor and playfulness, future work should investigate the emergence of humor and pretense across development and within the same context (i.e., social play). More research is also needed regarding whether and how specific types of play and humor support a child’s overall development (Bergen, 2019; Fink et al., 2020). In future work, it will be important to investigate links with creativity, social emotional adjustment, and cognitive skills (Bergen & Rousta, 2019; Rao & Gibson, 2019).

The present findings contribute to this endeavor by providing evidence for the theorized relationship between children’s tendency to produce humor and their social understanding skills (Bergen, 2002; Dunn, 1988; Leekam, 1991). Although in our previous work, we found that children’s humor was associated with their tendency to talk about internal states during the same play session (Paine et al., 2021), in the present study, we examined children’s spontaneous production of humor in play in relation to an independent battery of social understanding tasks.

These analyses showed that children’s sound play with siblings was associated with emotion understanding, both in terms of recognizing emotions in faces and understanding emotional perspectives. This is particularly interesting because sound play often co-occurs with other types of humor as a cue to signify humorous intent (the humor frame; Bergen, 1998, 2002; Garvey, 1977), as observed in previous studies (Paine, Howe, et al., 2019; Paine et al., 2021). In the present study, for example, one child presented their sibling with a toy wine glass and said, “Would you like a cup of teee-aaa?” in a low, gruff voice, to which their sibling laughed. This finding therefore aligns with Airenti’s (2016) suggestion that emotion understanding is particularly important for shared humor, when children recognize that the play partner understands and shares their playful intentions (Bariaud, 1989).

It was surprising, however, that more sophisticated forms of humor, such as word play and preposterous statements and humorous anecdotes, were not significantly associated with emotion understanding. Given that more sophisticated forms of emotion understanding emerge beyond the preschool years (Kramer & Lagattuta, 2022), our study provides a platform for future researchers to investigate humor production in relation to more diverse and complex dimensions of emotion understanding, such as knowledge about the causes and consequences of emotions, which emerges through middle childhood. We also found that children who engaged in playful teasing with their sibling during dress-up play were more likely to have made reference to cognitive states while playing alone with the Playmobil set (e.g., “Kate [Playmobil figure] does not know where it is.”). This finding corroborates and extends previous work showing that children’s humor production is associated with their propensity to refer to cognitive states within sibling interactions (Paine et al., 2021). Although our findings suggest that humor production is associated with thinking about others’ minds, the cross-sectional nature of these associations preclude us from making any conclusions about causality.

Given the links between sibling humor and the measures of social understanding, as well as the fact that children share humor in their earliest interactions (Mireault et al., 2011, 2014; Reddy & Mireault, 2015), it seems likely that humor with one’s sibling provides a context within which children come to understand the minds of others (Dunn, 1994). Indeed, the sibling relationship provides children with a “natural laboratory” to learn about the social and cognitive world (Howe & Recchia, 2014). With siblings, children can test out different forms of humor that may (or may not) elicit amusement without jeopardizing the relationship. Yet, children who have greater insight into the expectations, perspectives, and mental states of others may be more likely to produce humor that will prompt a positive response from their sibling. Indeed, Bariaud (1989) emphasized the importance of humor occurring within a frame of mutual playful intent; in the absence of this understanding, the sharing of incongruities can result in play breaking down (Paine et al., 2021; Semrud-Clikeman & Glass, 2010). In particular, the successful sharing of playful teasing—which could be upsetting if not understood or occurring outside of the humor frame—requires sensitivity to the mental states of others.

In some cases, sibling relationships may be a context where some children experience aggressive or derisive teasing that could be interpreted as negative or hurtful. In the present study, we did not try to identify these behaviors, as our overarching aim was to code playful incongruities, in line with definitions of humor set out in theory and literature (McGhee, 1979; Mireault & Reddy, 2016; Pien & Rothbart, 1976; Schultz, 1976). Therefore, we determined playful intentionality during sibling interactions by conservatively coding incongruities within the context of laughter. As such, the present study captures playful, light-hearted, positive dimensions of children’s emotional lives with their siblings in a naturalistic context.

However, humor can prompt disapproval and conflict (Paine et al., 2021), for example, where humor occurs outside of a frame of mutual playful intent (Semrud-Clikeman & Glass, 2010). Negative affect may also arise from aggressive teasing and antagonism. Given that sibling antagonism is negatively associated with theory of mind development (Song & Volling, 2018; Song et al., 2016), further research is required to delineate between humorous, light-hearted teasing and aggressive teasing in sibling relationships. Such investigations should record negative, as well as positive affective responses to these behaviors to capture the various affective dimensions of sibling relationships in relation to social–cognitive development.

### Gender and Gender Composition

Contrary to our expectations, we did not detect any differences in focal children’s production of humor according to their own gender or the dyadic gender composition or the age gap between themselves and their closest-in-age sibling. We did not detect gender differences in children’s production of humor, which contrasts with previous evidence that boys’ and girls’ humor production starts to diverge in middle childhood (McGhee, 1979; Paine, Howe, et al., 2019). However, evidence from other samples suggests that gender differences become more detectable in later childhood and adolescence (Wu et al., 2016).

Furthermore, the methods used for coding humor in this study may have been less sensitive to gender differences. Given that humor and laughter are related, but not synonymous (Mireault & Reddy, 2016; Singer, 2019), it is quite possible that our coding of humor within episodes that contained laughter—with the view to examine humor that was cued as playful and nonthreatening—underestimated boys’ humor production (i.e., by not capturing humorous bids that did not generate amusement from their siblings). Some evidence suggests that by middle childhood, boys become more frequent jokers (McGhee, 1979; Paine, Howe, et al., 2019) and adopt more aggressive styles of humor (James & Fox, 2018), while girls produce more laughter in response to other people’s humorous acts (McGhee, 1979). Given observations that brothers may use humor in the context of conflict (Paine, Howe, et al., 2019), future studies are needed to clarify the role of gender composition in sibling pairs in successful and unsuccessful humorous exchanges during contentious interactions.

Furthermore, sex and gender have differential effects on humor in different cultural contexts (Chen & Martin, 2007). As such, the discrepancy with earlier work may also reflect ways in which humor tends to be socialized in family homes in different cultures. An interesting avenue for future research would therefore be to investigate how humor is shared in interactions that involve other family members, and how humor is encouraged or, potentially, rebuked by caregivers in polyadic interactions (i.e., involving ≥ 3 family members), in different sociocultural contexts (Persram et al., 2019).

### Limitations

Although the analyses drew on a large battery of assessments and observations in a moderately sized sample, this study has some limitations. It will be important for future researchers to examine children’s use of humor in different sociocultural contexts, in samples representing different birth orders and only children, and in the context of other child-child relationships. The present study’s overarching aim was to examine associations between humor production in middle childhood and social understanding skills. Thus, our analyses focused on the older sibling, who was the focal child in a longitudinal study. However, we found relatively small associations between children’s humor production and within-child characteristics. Rather, as in previous studies of siblings, humor production was highly dyadic (Paine, Howe, et al., 2019; Paine et al., 2021). This emphasizes the importance of the social interactional context when studying correlates of children’s behavior in play (Gibson et al., 2020), which should inform future work on humor. A more comprehensive, enriched analysis of actor and partner effects would be an interesting avenue for future research (Kenny et al., 2006). Such analyses could elucidate dyadic patterns of how siblings share humor during play and identify potential correlates of different dyadic patterns (e.g., sibling relationship quality).

The few significant associations detected between humor production and pretense may indicate that both are distinct constructs that should be studied in relation to children’s developing social understanding. However, given that we harnessed data from an existing dataset that was not primarily designed to study humor and pretend play, our findings may also be limited by the length of the observations. For example, although most children engaged in pretend enactments, we observed that few children engaged in creative object use (i.e., transforming the identity or function of an object) during the free play task with Playmobil. Similarly, although most children produced humor with their sibling, we observed that some types of humor were produced by a small minority of children. Possibly, longer observations would have given children more opportunities to engage in creative pretend play and humor, yet we note that in longer observations of social play, children tend to spend a great deal of time “setting up” and organizing play rather than engaging in pretense (Gibson et al., 2020; Howe et al., 2014). Given it is possible that experimenter presence may have inhibited the occurrence of humor and creative pretense, future researchers may also consider conducting observations without experimenters present.

## Conclusion

It is well known that sibling relationships are an important context for developing an understanding of the social and mental world. Yet children’s humor with siblings and how it relates to their developing understanding of minds has long been overlooked. The present findings expand our knowledge of humor production as it relates to children’s engagement in pretense and demonstrates that humor with siblings provides a window to social understanding in childhood. In line with social interactionist theories of development, our findings suggest that humor in play, like social pretend play, provides children with a context for rehearsing existing and developing new social understanding skills (Gibson et al., 2020; Lillard et al., 2013). These findings provide a platform for future investigators to investigate further the social, cognitive, and emotional functions of humor in child development.

## Supplementary Material

10.1037/dev0001403.supp

## Figures and Tables

**Table 1 tbl1:** Sociodemographic Characteristics of the Sample at Entry to the Study

	Sibling play sample
Characteristic	(*N* = 110)
Mother’s age in years at first birth, *M* (*SD*), range	29.17 (5.71), 16.09−40.38
Cultural identity: Welsh, English, Scottish, Irish	98.1%
Achieved 5 GCSEs A-C or equivalent+ or higher	84.5%
Married or cohabiting	90.9%
Social class (middle class)	62.7%
Participating child’s gender (female)	46.4%
*Note*. % indicates percent of sample. +Completed secondary education (age 16).	

**Table 2 tbl2:** Humor Coding Scheme Applied to 10-s Intervals of Sibling Interaction Containing Laughter

Humor categories	Examples
a. Performing incongruities: Enacting a conflict between what is normal/expected and reality with an object. For example, placing an object in a wrong location or making a toy perform a wrong action.	FC puts teacup on head. FC throws their own hat against the wall.
b. Word play: Nonsense words, rhyming words, riddles, jokes, label-based humor. Making deliberate mistakes in language or changing words in well-known songs.	FC says, “Wait a minute, this is hotsie totsie.” FC “A pirate goes nee-nor-nee-nor!”
c. Preposterous statements and humorous anecdotes: Creating absurd or unusual stories, anecdotes, or making announcements, nonsense sentences, deliberate falsehoods (identified by conflicting statements).	FC “I’m arrested for eating cake!” FC “[Robbers] steal money and jewels and… bears!”
d. Sound play: Over exaggerated vocalizations or speech, exaggerated gasps, animal noises, using a very deep or gruff voice in a silly or unconventional way (e.g., fast or slow), or using silly accents, chanting, bursting into exaggerated song.	FC (Singing) “Oh a-ding-ding!” FC (Squeaky voice while waving) “Hello hello!”
e. Taboo: Disgusting noises, such as blowing raspberries, fart noises, burp noises, using taboo words or discussion and/or enacting taboo themes. Includes violent themes of play, like stabbing, shooting, or terms like “die!” Any play that is rule breaking (yet playful) in nature.	FC pretends to pour into a teacup, then “throws” the tea in younger sibling’s face. FC uses play knife to “saw” sibling’s arm off.
f. Playful teasing: Light-hearted, playful, mischievous behavior directed to play partner. Includes light-hearted insults and playful rough and tumble. Must be coupled with playful cues (smiling, laughter, playful tone of voice).	FC “[Sibling name] gets the saucer and I get the teapot so [Sibling name] can’t have any tea!” FC, smiling, steals hat from sibling.
g. Clowning: Silly or over exaggerated body movements, dancing, posing or pulling funny faces.	FC pokes tongue out at sibling. FC jumps around the room like a frog.
*Note*. Categories could co-occur. FC = focal child.	

**Table 3 tbl3:** Means, Standard Deviations, and Ranges of Raw Categories and Total Humor Production During Dress-Up Play With a Sibling

Humor category	*M* (*SD*)	Range	%
Performing incongruities	1.51 (2.44)	0–13	55.5
Word play	0.23 (0.55)	0–2	16.4
Preposterous statements and humorous anecdotes	0.52 (1.06)	0–7	30.9
Sound play	0.38 (0.86)	0–5	23.6
Taboo	0.38 (1.48)	0–13	12.7
Playful teasing	0.49 (1.03)	0–6	25.5
Clowning	0.16 (0.66)	0–6	10.9
Total humor	3.67 (5.18)	0–36	75.5
*Note*. *N* = 110. % refers to percentage of children who demonstrated the behavior at least once during the play session.			

**Table 4 tbl4:** Bivariate Correlations Between Focal Children’s Humor Production in Play With a Sibling and Pretend Play and Social Understanding

	Solo pretend play with Playmobil		Social understanding		
Children’s humor with siblings in the dressing-up task	Setting up objects	Expected and creative use of objects	Second-order false belief	Children’s references to others’ cognitions in solo Playmobil play	Emotion understanding
Performing incongruities	.04	.09	−.15	.08	−.04
Word play	−.10	.23*	−.10	.14	.04
Preposterous statements and humorous anecdotes	.04	.02	.04	.08	.16^+^
Sound play	−.13	.07	.08	.19*	.22*
Taboo	−.11	.12	−.02	.12	.03
Playful teasing	.05	−.03	.00	.26**	.12
Clowning	−.12	.14	−.05	.03	.14
Total humor	−.03	.14	−.08	.18^+^	.09
*N*	105	105	108	106	109
*Note*. Pearson correlations.^+^ *p* < .10.					
* *p* < .05. ** *p* < .01.					

**Table 5 tbl5:** Prediction of Types of Humor Focal Children Produced When in Play With Their Sibling

Predictor	Δ*R*^2^	B (*SE*)	β	95% CI for B
Model 1				
Child receptive vocabulary		.001 (.002)	−.07	[−.004, .002]
Child working memory	.002	.000 (.001)	−.02	[−.002, .002]
Child emotion understanding	.05*	.004 (.002)	.24*	[.001, .007]
Constant		−.11 (.18)		[−.47, .26]
Model 2				
Child talkativeness in solo Playmobil play	.03	.08 (.07)	.11	[−.07, .23]
Child references to others’ cognitions in solo Playmobil play	.02	.07 (.04)	.15	[−.02, .15]
Constant		.03 (.05)		[−.07, .12]
Model 3				
Child talkativeness in solo Playmobil play	.03	.08 (.05)	.10	[−.09, .25]
Child references to others’ cognitions in solo Playmobil play	.04*	.11 (.09)	.22*	[.01, .21]
Constant		.04 (.05)		[−.07, .15]
*Note*. Models 1 and 2 predict sound play with a sibling in middle childhood, and Model 3 predicts playful teasing with a sibling in middle childhood. Coefficients presented are those obtained in the final models.					
* *p* < .05.					
