# Supplementary material for: “A Pirate Goes Nee-Nor-Nee-Nor!” Humor With Siblings in Middle Childhood: A Window to Social Understanding?
Source: Dev Psychol. 2022 Jun 2;58(10):1986–98. doi: 10.1037/dev0001403 (PMC9494906; doi:10.1037/dev0001403)
Supplement: Supplementary file 1 [file DEV-2021-4578_Supplemental_Materials.docx]

|  | 1. | 2. | 3. | 4. | 5. | 6. | 7. | 8. | 9. | 10. | 11. | 12. | 13. | 14. | 15. | 16. | 17. | 18. |
| --- | --- | --- | --- | --- | --- | --- | --- | --- | --- | --- | --- | --- | --- | --- | --- | --- | --- | --- |
| 1. Total humor | - | .82^**^ | .50^**^ | .45^**^ | .53^**^ | .52^**^ | .60^**^ | .41^**^ | .02 | -.11 | -.05 | .05 | .00 | -.03 | .14 | .18^+^ | .09 | .09 |
| 1. Performing incongruities |  | - | .31^**^ | .25^**^ | .29^**^ | .39^**^ | .36^**^ | .21^*^ | -.05 | -.04 | -.09 | .07 | .06 | .04 | .09 | .08 | .05 | -.04 |
| 1. Word play |  |  | - | .13 | .35^**^ | .31^**^ | .26^**^ | .48^**^ | .00 | -.12 | .08 | .04 | .08 | -.10 | .23* | .14 | .03 | .04 |
| 1. Preposterous statements and humorous anecdotes |  |  |  | - | .18^+^ | -.02 | .18^+^ | -.11 | .07 | .02 | .16 | .05 | -.05 | .04 | .02 | .08 | .04 | .16^+^ |
| 1. Sound play |  |  |  |  | - | .30^**^ | .23^*^ | .41^**^ | .11 | -.02 | .03 | .05 | .03 | -.13 | .07 | .19^*^ | .17^+^ | .22^*^ |
| 1. Taboo |  |  |  |  |  | - | .47^**^ | .33^**^ | -.01 | -.12 | -.06 | .11 | .15 | -.11 | .12 | .12 | .10 | .03 |
| 1. Banter |  |  |  |  |  |  | - | .29^**^ | .09 | -.06 | -.01 | .03 | -.04 | .05 | -.03 | .26^**^ | .18^+^ | .12 |
| 1. Clowning |  |  |  |  |  |  |  | - | .14 | -.11 | .06 | .11 | .08 | -.12 | .14 | .03 | .06 | .14 |
| 1. Child age^a^ |  |  |  |  |  |  |  |  | - | -.25^*^ | .27^**^ | -.12 | -.17^+^ | -.01 | .07 | -.02 | -.04 | .17+ |
| 1. Child receptive vocabulary (BPVS) |  |  |  |  |  |  |  |  |  | - | .14 | .21^*^ | .40^**^ | .16 | -.12 | .07 | .15 | .29^**^ |
| 1. Child working memory (ANT) |  |  |  |  |  |  |  |  |  |  | - | .14 | .21^*^ | .11 | .03 | -.11 | -.06 | .27^**^ |
| 1. Maternal social class |  |  |  |  |  |  |  |  |  |  |  | - | .50^**^ | -.06 | -.01 | .18^+^ | .07 | .09 |
| 1. Maternal education |  |  |  |  |  |  |  |  |  |  |  |  | - | .10 | .02 | .04 | .15 | .15 |
| 1. Child setting up objects in solo Playmobil^®^ play |  |  |  |  |  |  |  |  |  |  |  |  |  | - | -.64^**^ | -.05 | -.27^**^ | .05 |
| 1. Child expected and creative use of objects in solo pretense with Playmobil^®^ |  |  |  |  |  |  |  |  |  |  |  |  |  |  | - | -.06 | .19^+^ | .01 |
| 1. Child references to others’ cognitions in solo Playmobil^®^ play |  |  |  |  |  |  |  |  |  |  |  |  |  |  |  | - | .36^**^ | .08 |
| 1. Child talkativeness in solo Playmobil^®^ play |  |  |  |  |  |  |  |  |  |  |  |  |  |  |  |  |  | .04 |
| 1. Emotion understanding |  |  |  |  |  |  |  |  |  |  |  |  |  |  |  |  |  | - |
